# Supplementary figures and images for: Trends and patterns of antibiotic prescribing at orthopedic inpatient departments of two private-sector hospitals in Central India: A 10-year observational study
Source: PLoS One. 2021 Jan 27;16(1):e0245902. doi: 10.1371/journal.pone.0245902 (PMC7840031; doi:10.1371/journal.pone.0245902)

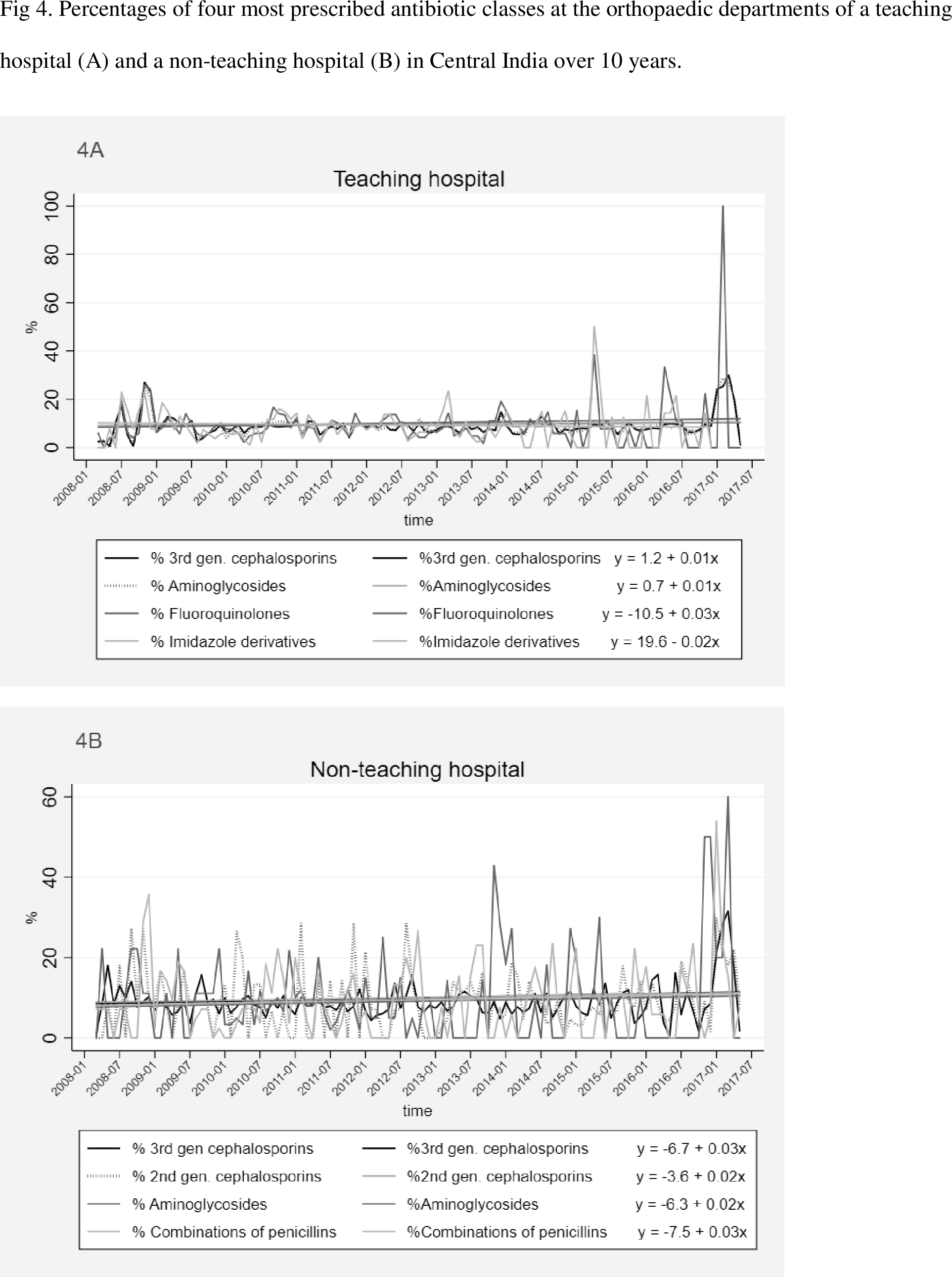

Supplement: S1 Fig — (TIF) [file pone.0245902.s001.tif]

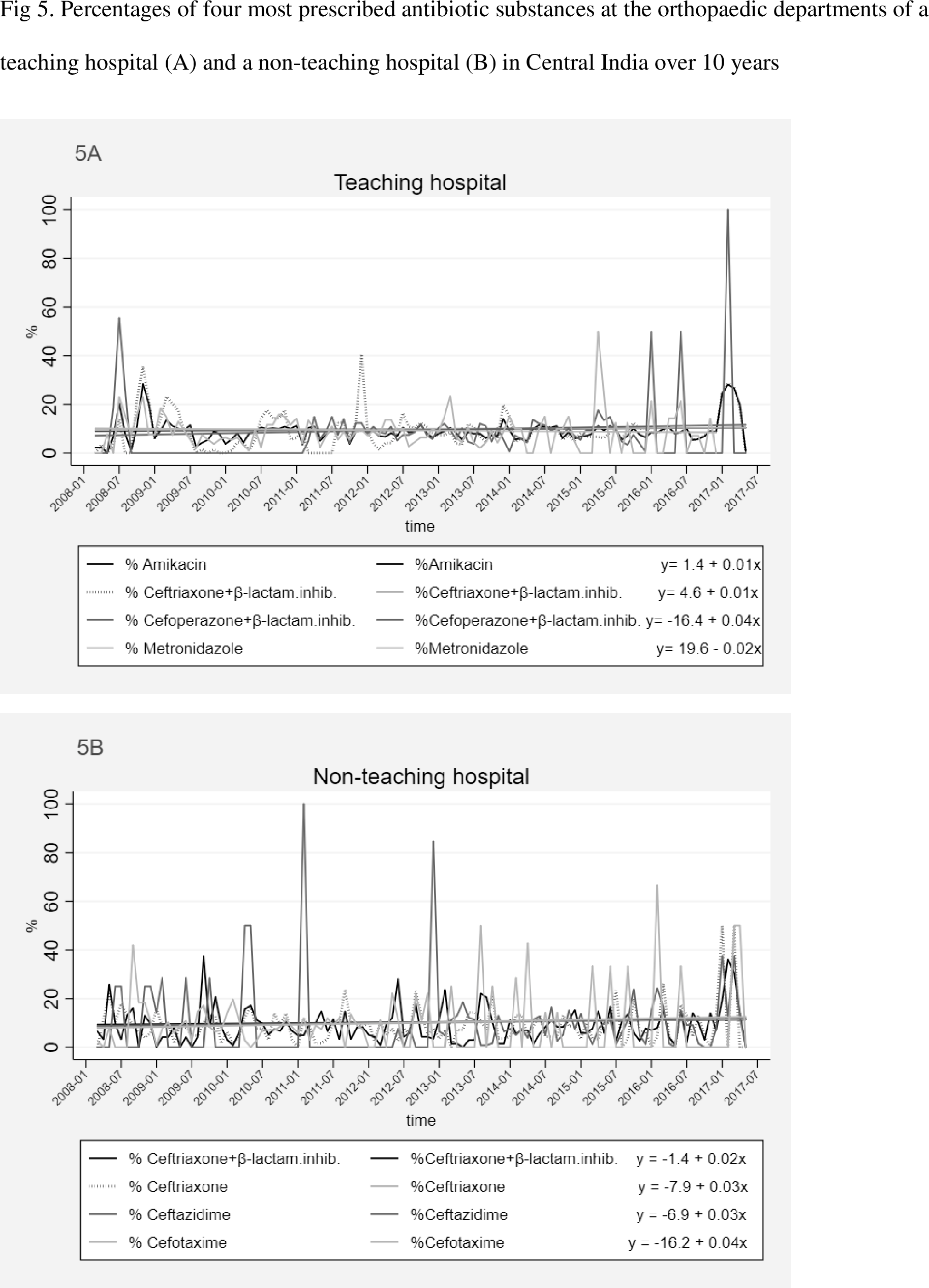

Supplement: S2 Fig — (TIF) [file pone.0245902.s002.tif]
