# Supplementary material for: Trends and patterns of antibiotic prescribing at orthopedic inpatient departments of two private-sector hospitals in Central India: A 10-year observational study
Source: PLoS One. 2021 Jan 27;16(1):e0245902. doi: 10.1371/journal.pone.0245902 (PMC7840031; doi:10.1371/journal.pone.0245902)
Supplement: S1 Appendix — (DOCX) [file pone.0245902.s003.docx]

**Appendix A: Comparison of prescription trends of four most prescribed antibiotic classes**

In the TH, the most prescribed antibiotic classes were 3^rd^ generation cephalosporins (J01DD, 38%), other aminoglycosides (J01GB, 32%), fluoroquinolones (J01MA, 9%) and imidazole derivatives (J01XD, 5%). The prescription of 3^rd^ generation cephalosporins, other aminoglycosides and fluoroquinolones had significant increasing trends, among which the prescription of fluoroquinolones increased the most over 10 years (β=0.03, p<0.001, Fig 4A). On the other hand, the prescription of imidazole derivatives significantly decreased over time (β= -0.02, p<0.001, Fig 4A).

In the NTH, the most prescribed antibiotic classes were: 3^rd^ generation cephalosporins (J01DD, 66%), 2nd generation cephalosporins (J01DC, 14%), other aminoglycosides (J01GB, 5%) and combinations of penicillins, including β-lactamase inhibitors (J01CR, 5%). In the NTH, the prescription of all the most prescribed antibiotic classes significantly increased over 10 years, with 3^rd^ generation of cephalosporins and combinations of penicillins including β -lactamase inhibitors having the highest rates of increase (β=0.03, p<0.001, Fig 4B). The most frequently prescribed antibiotic class in both hospitals, 3^rd^ generation cephalosporins, had three times higher rate of increase in the NTH (β=0.03, p<0.001, Fig 4B) compared to the TH (β=0.01, p<0.001, Fig 4A).

**Fig 4. Percentages of four most prescribed antibiotic classes at the orthopaedic departments of the teaching hospital (A) and the non-teaching hospital (B) in Central India over 10 years.**
