# Supplementary material for: Trends and patterns of antibiotic prescribing at orthopedic inpatient departments of two private-sector hospitals in Central India: A 10-year observational study
Source: PLoS One. 2021 Jan 27;16(1):e0245902. doi: 10.1371/journal.pone.0245902 (PMC7840031; doi:10.1371/journal.pone.0245902)
Supplement: S2 Appendix — (DOCX) [file pone.0245902.s004.docx]

**Appendix B: Comparison of prescription trends of the four most frequently prescribed antibiotic substances**

In the TH, the most prescribed antibiotics were: amikacin (J01GB06, 31%**),** ceftriaxone and β-lactamase inhibitor (J01DD63, 16%), cefoperazone and β-lactamase inhibitor (J01DD62, 7%) and metronidazole (J01XD01, 5%). The usage of these antibiotics significantly increased over time, except for metronidazole, whose prescription decreased (β= -0.02, p<0.001, Fig 5A). The prescription of cefoperazone and β-lactamase inhibitor had the highest increase over 10 years (β=0.04, p<0.001, Fig 5A).

In the NTH, the most prescribed antibiotics were: ceftriaxone and β-lactamase inhibitor (J01DD63, 22%), ceftriaxone (J01DD04, 14%), ceftazidime (J01DD02, 11%) and cefotaxime (J01DD01, 10%). The trends of all the most prescribed antibiotics in the NTH significantly increased over 10 years, with cefotaxime having the highest rate of increase (β=0.04, p<0.001, Fig 5B).

**Fig 5. Percentages of four most prescribed antibiotic substances at the orthopaedic departments of the teaching- (5A) and the non-teaching hospital (5B) in Central India over 10 years.**
