# Supplementary material for: Trends and patterns of antibiotic prescribing at orthopedic inpatient departments of two private-sector hospitals in Central India: A 10-year observational study
Source: PLoS One. 2021 Jan 27;16(1):e0245902. doi: 10.1371/journal.pone.0245902 (PMC7840031; doi:10.1371/journal.pone.0245902)
Supplement: Attachment — Submitted filename: Reviewer Comments PONE 2020.docx [file pone.0245902.s005.docx]

Reviewer Comments PONE-D-20-33344

Trends and patterns of antibiotic prescribing at orthopedic inpatient departments of two

private-sector hospitals in Central India: A 10-year observational study

Authors have studied an important area of antibiotic prescribing in orthopaedic in-patients in two private set ups in India. My specific comments are as follows

1. Page 9 Line 138: The comparison was based on the presence of prescribed antibiotics in the latest available versions of the National List of Essential Medicines India (NLEMI, 2015) and the WHO Model List of Essential Medicines (WHOMLEM, 141 2019).

Line 179: Adherence to the WHOMLEM was higher than adherence to the NLEMI in both hospitals.

There is a difference in the adherence. What may be the causes?

2. There is a wide difference in antibiotics prescribed by generic name and brand name between the 2 hospitals. What is the reason?
3. Line 272: However, it is noteworthy that fractures are not necessarily indications for prescription of antibiotics, unless: a) there is a presence of open or multiple fractures that present a greater risk for infection; b) there is associated infectious diagnosis;

Is there a break up of these a, and b, in the two hospital? OR is there a difference in the study participants between the two hospitals, as far as diagnosis is compared.

4. 232: first repeated – spelling mistake? May be it should be ‘first reported’
5. It will be a better idea to classify antibiotic usage as per “AWARE” category-Access, Watch and Restricted class. This will give an idea which class of drugs were trending.

Is there increase in Access class/ Watch class/Restricted class of antibiotics?

6. There is a general increase in antibiotic prescription in TH and NTH; But the rate of change is higher in NTH.

This change is similar to increase in number of fracture cases.

However,

Is there an increase in operations performed?/ or is there any trend?

Is there an increase in length of hospital stay/ or is there any trend?

Is there any change in mortality/survival?
